# Schiff Base-Functionalized Melamine Sponge with Hierarchical Porous Architecture for High-Efficiency Removal of Organic Dyes in Wastewater

**DOI:** 10.3390/nano15151157

**Published:** 2025-07-26

**Authors:** Xiaoyu Du, Hailiang Nie, Yanqing Qu, Jingyu Xu, Hongge Jia, Yong Zhang, Wenhui Ma, Boyu Du

**Affiliations:** 1 Heilongjiang Provincial Key Laboratory of Polymeric Composition, College of Chemistry and Chemical Engineering, Qiqihar University, Qiqihar 161006, China; 2 Guangxi Key Laboratory of Clean Pulp & Papermaking and Pollution Control, Guangxi University, Nanning 530004, China

**Keywords:** melamine foam, composite foam, absorption, Congo red dye

## Abstract

Melamine sponges have demonstrated significant application potential in the field of adsorption materials due to their unique three-dimensional porous network structure, excellent chemical/mechanical stability, and abundant amino active sites on the surface. However, the development of modified melamine sponges with efficient Congo red dye removal capabilities remains a substantial challenge. In this study, a stable linear polymer network structure was constructed on the surface of melamine sponges via an in situ polymerization strategy based on the Schiff base reaction mechanism. Characterization analyses reveal that the modified sponge not only retained the original porous skeleton structure but also significantly enhanced the density of surface active sites. Experimental data demonstrate that the modified sponge exhibited excellent adsorption performance for Congo red dye, with the adsorption process conforming to the pseudo-second-order kinetic model and achieving a practical maximum adsorption capacity of 380.4 mg/g. Notably, the material also displayed favorable cyclic stability. This study provides an efficient adsorbent for Congo red dye-contaminated wastewater treatment through the development of a novel surface-functionalized sponge material while also offering new solutions for advancing the practical applications of melamine-based porous materials and environmental remediation technologies.

## 1. Introduction

In the context of rapid industrial development, dyes play a pivotal role across numerous sectors, including printing and dyeing, textiles, paper-making, pharmaceuticals, and food processing [1,2]. However, their extensive utilization has raised significant environmental concerns, particularly regarding organic dyes characterized by high water solubility, inherent toxicity, and carcinogenic properties [3,4,5,6]. Dye-laden wastewater typically contains complex organic compounds such as azo derivatives, which exhibit exceptional chemical stability and resistance to natural degradation, enabling their persistent accumulation in ecosystems. As a representative azo dye, Congo red (CR) is a benzidine-derived anionic disodium salt featuring an azo group (-N=N-) conjugated with aromatic moieties at both termini [7,8]. This sophisticated architecture endows CR with remarkable thermal stability, pronounced resistance to microbial degradation, and photochemical inertness. Consequently, CR-contaminated effluents are frequently associated with strongly acidic conditions and elevated heavy metal concentrations, generating synergistic toxic effects in aquatic ecosystems while demonstrating exceptional recalcitrance to conventional remediation approaches [9]. Such contamination severely impairs photosynthetic processes in aquatic flora and compromises the survival of aquatic biota while posing substantial carcinogenic and mutagenic risks to humans and wildlife. Given these critical challenges, the development of effective strategies for CR removal from wastewater systems has emerged as an urgent priority in environmental engineering and pollution control research [10].

In recent years, the environmental science and engineering communities have developed diverse remediation technologies for eliminating hazardous dyes such as CR from industrial effluents. Current research primarily focuses on advancing three principal strategies: photocatalytic [11], membrane separation [12], and adsorption [13,14]. While these approaches demonstrate varying degrees of effectiveness in aquatic pollutant management, comprehensive techno-economic analyses and practical implementation assessments have established adsorption as the most promising scalable solution for dye removal, owing to its operational simplicity and cost-efficiency [14,15,16]. The efficacy of adsorption processes predominantly hinges on critical adsorbent characteristics, including specific surface area, pore architecture, and surface functionalization [17]. Consequently, the design of novel adsorbent materials combining high adsorption capacity, exceptional chemical stability, favorable regeneration potential, and economic viability has emerged as a pivotal research frontier in water treatment engineering. Earlier investigations predominantly explored modification strategies for conventional adsorbents like activated carbon, bentonite, and diatomite. However, inherent limitations such as poor adsorption selectivity, limited capacity, and challenging recovery processes have significantly constrained their practical application in real-world wastewater treatment scenarios [18].

Melamine sponge (MS), a low-cost and commercially available cleaning material [19], has shown significant potential in pollutant adsorption due to its three-dimensional porous structure and abundant surface amino functional groups [20,21]. Compared to conventional adsorbents, its advantages are manifested in the following aspects: First, the porous network structure provides a high specific surface area and efficient mass transport channels [22]; second, surface amino groups directly serve as active sites, avoiding complex surface modification procedures; third, its mechanical flexibility and chemical stability enable applicability in complex environments [23]. Recent studies have expanded its functional applications through various modification strategies. For heavy metal adsorption, Feng et al. [24] coated melamine sponge with sodium alginate, significantly enhancing Pb^2+^ adsorption performance via synergistic coordination between amino and carboxyl groups. The Lan group [25] loaded magnetic hydroxyapatite nanoparticles onto the sponge, retaining its porous structure while endowing magnetic separation capability, achieving efficient Pb^2+^ removal and recyclability. For anionic organic pollutants, Amaly et al. [26] covalently modified the sponge with glycidyltrimethylammonium chloride, producing a polycationic material that effectively adsorbs anionic dyes through electrostatic interactions. Additionally, Li et al. [27] developed an MF@COF composite by in situ growth of heteropore covalent organic frameworks (COFs) on the melamine sponge surface, creating a superhydrophobic material with hierarchical porosity. This composite demonstrated ultrahigh adsorption capacity and rapid kinetics for oil pollutants, highlighting the promise of melamine sponge in oil–water separation applications [28].

**Figure 1:**
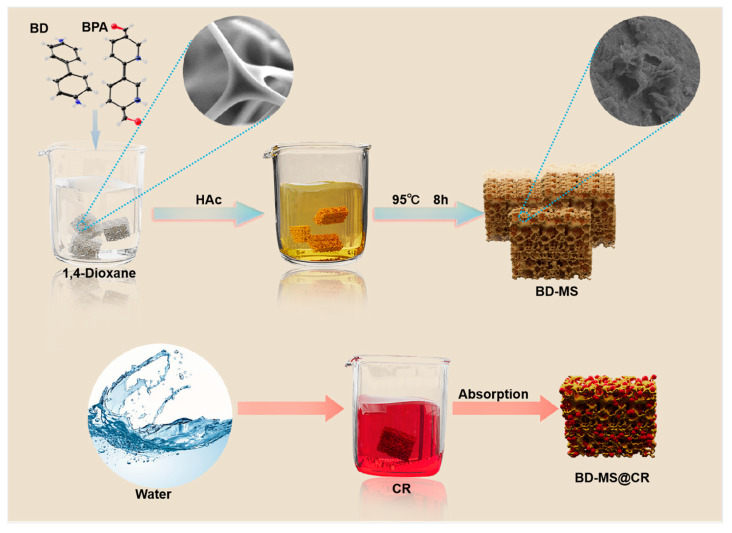
Synthesis process of BD-MS and its adsorption capacity for CR.

Building upon the superior adsorption performance of melamine sponge, this study employs a novel surface modification strategy. The three-dimensional porous architecture of melamine sponge inherently provides a high specific surface area, while its framework-rich -NH_2_ groups serve as functional sites for polymer growth. Through the in situ controlled polymerization of benzidine and 2,2′-bipyridine−5,5′-dicarboxaldehyde on a melamine sponge framework, we achieved linear polymer growth while preserving the integrity of the substrate’s three-dimensional structure. This enabled the construction of a modified melamine sponge composite adsorbent (BD-MS), thereby endowing it with novel surface properties. (Figure 1). Throughout the synthesis process, BD-MS utilizes the three-dimensional (3D) sponge skeleton as a support, enabling uniform polymer coverage. This composite material not only completely retains the inherent high specific surface area and 3D porous skeletal structure of the pristine sponge but also exhibits enhanced thermal stability and excellent reproducibility. More importantly, the surface polymer layer facilitates efficient and rapid adsorption of dye molecules with high adsorption capacity through the synergistic effects of hydrogen bonding, π-π interactions, and electrostatic interactions. To systematically characterize the physicochemical attributes of the modified material, X-ray diffraction (XRD) and Fourier-transform infrared spectroscopy (FT-IR) were employed to verify structural modifications, thermogravimetric analysis (TGA) was used to evaluate thermal stability, and Brunauer–Emmett–Teller (BET) surface area analysis was utilized to investigate porous structure evolution. Furthermore, adsorption performance toward CR dye was rigorously examined through the establishment of kinetic and thermodynamic models.

## 2. Experiment

### 2.1. Materials

Benzidine (≥98%), 2,2′-bipyridine-5,5′-dicarbaldehyde (≥98%), glacial acetic acid (99.5%), and 1,4-dioxane (ACS, ≥98%) were obtained from Aladdin Chemistry Co., Ltd. (Shanghai, China). Methylene blue (MB), rhodamine B (RhB), malachite green (MG), methyl orange (MO), and Congo red (CR) were purchased from Aladdin Chemistry Co., Ltd. (Shanghai, China). Melamine sponge was purchased from a local supermarket. The following chemicals were supplied by Tianjin Kemio Chemical Reagent Co., Ltd. (Tianjin, China): ethanol (99%), tetrahydrofuran (99%), NaOH, and HCl solution (38 wt%). Deionized (DI) water used in all investigations was produced in our laboratory. All purchased pharmaceuticals and solvents were employed without additional purification.

### 2.2. Synthesis and Modification of Sponge Composite Materials

The fabrication of modified melamine sponge with tailored properties was achieved through an optimized one-pot solvothermal method adapted from previous studies [29]. Initially, bulk white melamine sponge was sectioned into cubic specimens (1 cm^3^). These cubes were sequentially subjected to ultrasonic cleaning in deionized water and anhydrous ethanol (30 min each), followed by overnight drying at 60 °C. Subsequently, 2,2′-bipyridine-5,5′-dicarbaldehyde (BPA, 0.125 mol) was dissolved in 15 mL of 1,4-dioxane, followed by immersion of three pretreated sponge cubes. The mixture underwent 15 min of ultrasonication to ensure homogeneous BPA dispersion. Acetic acid (1 mL) was then introduced, with continued ultrasonication for 15 min, followed by the addition of benzidine (BD, 0.125 mol) and another 15 min of ultrasonication to optimize reactant interaction. The reaction system was maintained at 95 °C for 10 h. Post-synthesis, the samples were washed three times with tetrahydrofuran (THF) and ethanol (EtOH), respectively, to remove unreacted precursors and byproducts and then vacuum-dried at 60 °C for 12 h to yield the modified sponge. The surface of the modified sponge displayed a yellowish hue and an increased roughness. The preparation procedure is illustrated in Figure 1.

### 2.3. Characterization

This study systematically analyzed the material using integrated characterization techniques. X-ray diffraction (XRD) measurements were conducted on a Rigaku SmartLab SE diffractometer with Cu Kα radiation (λ = 1.5406 Å), scanning the 2θ range of 5–40° at a rate of 2°/min. X-ray photoelectron spectroscopy (XPS) analysis was performed using a Thermo Scientific K-Alpha spectrometer equipped with monochromatic Al Kα X-rays under an ultrahigh vacuum of 10^−7^ mbar. Fourier-transform infrared (FTIR) spectra were obtained in attenuated total reflectance (ATR) mode on a Thermo Nicolet iS20 spectrometer, covering the wavenumber range of 4000–400 cm^−1^. Thermogravimetric analysis (TGA) was carried out on a Rigaku TG/DTA 8122 thermal analyzer under a nitrogen atmosphere, heating from 30 °C to 800 °C at 10 °C/min. The specific surface area and pore characteristics were determined via N_2_ adsorption–desorption experiments at 77 K using a Micromeritics ASAP 2460 analyzer, with samples pretreated by vacuum degassing at 150 °C. Microstructural morphology was characterized by a ZEISS GeminiSEM 300 field-emission scanning electron microscope (FE-SEM) operated at 1–5 kV for high-resolution imaging. Ultraviolet-visible (UV–Vis) spectra were recorded on a UV-5500PC spectrophotometer (Shanghai Yuanxi Instrument Co., Ltd., Shanghai, China).

### 2.4. CR Adsorption and Removal by BD-MS

This study systematically investigated the adsorption performance through the following protocol: Typically, 8 mg of BD-MS was submerged in 15 mL of CR aqueous solutions with varying initial concentrations (50–300 ppm), and the mixture was maintained at room temperature in the dark under continuous stirring for 24 h. After adsorption, the MS was removed from the solution using tweezers to separate the adsorbent. Subsequently, the equilibrium concentration of CR in the supernatant was determined at its maximum absorption wavelength (λ_max_ = 496 nm) using a UV-Vis spectrophotometer. The removal efficiency (R) and adsorption capacity (Q_e_) of MS toward CR were calculated according to Equations (1) and (2), respectively [30]:

(1) $$R\left(\%\right)=\frac{\left(C_{0}-C_{e}\right)}{C_{0}}\times 100$$

(2) $$Q_{e}=\frac{V(C_{0}-C_{e})}{m}$$

In this context, C_0_ and C_e_ (mg·L^−1^) represent the initial and equilibrium concentrations of the dye in the aqueous solution, respectively; V (mL) denotes the volume of the dye solution, and m (g) is the mass of the BD-MS sample. Furthermore, to investigate the adsorption behavior of BD-MS toward CR, the equilibrium adsorption isotherms [31,32] were fitted using the Langmuir, Freundlich, Temkin, and Sips models, with the corresponding Equations (3)–(6):

(3) $$Q_{e}=\frac{Q_{m}\cdot K_{L}\cdot C_{e}}{1+K_{L}\cdot C_{e}}$$

(4) $$Q_{e}=K_{F}\cdot C_{e}^{1/n}$$

(5) $$Q_{e}=B\ln A+B\ln C_{e}$$

(6) $$Q_{e}=\frac{{Q_{m}(K_{s}\cdot C_{e})}^{n}}{1+{\left(K_{s}\cdot C_{e}\right)}^{n}}$$

Here, C_e_ (mg·L^−1^) represents the equilibrium concentration of the dye in the aqueous solution, Q_e_ (mg·g^−1^) denotes the equilibrium adsorption capacity, and Q_m_ (mg·g^−1^) corresponds to the maximum adsorption capacity. In the adsorption isotherm equations, K_L_ is the Langmuir constant, K_F_ is the Freundlich constant, 1/n is the adsorption intensity, B is the factor related to adsorption heat, A is the equilibrium binding constant, K_s_ is the Sips equilibrium constant, and n is the heterogeneity parameter. For the kinetic adsorption study, a similar methodology was adopted: 8.0 mg of BD-MS was added to 15 mL of CR aqueous solution, and the dye concentration was measured at predetermined time intervals. The kinetic data were fitted using the pseudo-first-order, pseudo-second-order, Elovich, and intraparticle diffusion models, represented by Equations (7), (8), (9), and (10), respectively [33,34]:

(7) $$q_{t}=q_{e}(1-e^{-k_{1}t})$$

(8) $$q_{t}=\frac{k_{2}q_{e}^{2}t}{1+k_{2}q_{e}t}$$

(9) $$q_{t}=\frac{1}{\beta }ln(\alpha \beta t-1)$$

(10) $$q_{t}=k_{i}t^{0.5}+C$$

For selective adsorption studies, 8.0 mg of BD-MS was added to 15 mL aqueous solutions of methylene blue (MB), rhodamine B (RhB), malachite green (MG), and methyl orange (MO). The mixtures were stirred for 24 h at room temperature under dark conditions. Subsequently, the modified sponge was collected, and the equilibrium concentrations of MB, RhB, MG, and MO in the post-adsorption solutions were determined at their respective maximum absorption wavelengths (λ_max_ = 664 nm for MB, λ_max_ = 554 nm for RhB, λ_max_ = 618 nm for MG, λ_max_ = 464 nm for MO) using a UV-Vis spectrophotometer. For pH-dependent adsorption investigations of CR on BD-MS, solution pH was adjusted with dilute NaOH (0.1 M) and HCl (0.1 M) solutions. After 24 h of adsorption under dark conditions at room temperature, the equilibrium concentration of CR was determined through UV-vis spectrophotometric analysis.

## 3. Results and Discussion

### 3.1. Synthesis and Characterization of BD-MS

**Figure 2:**
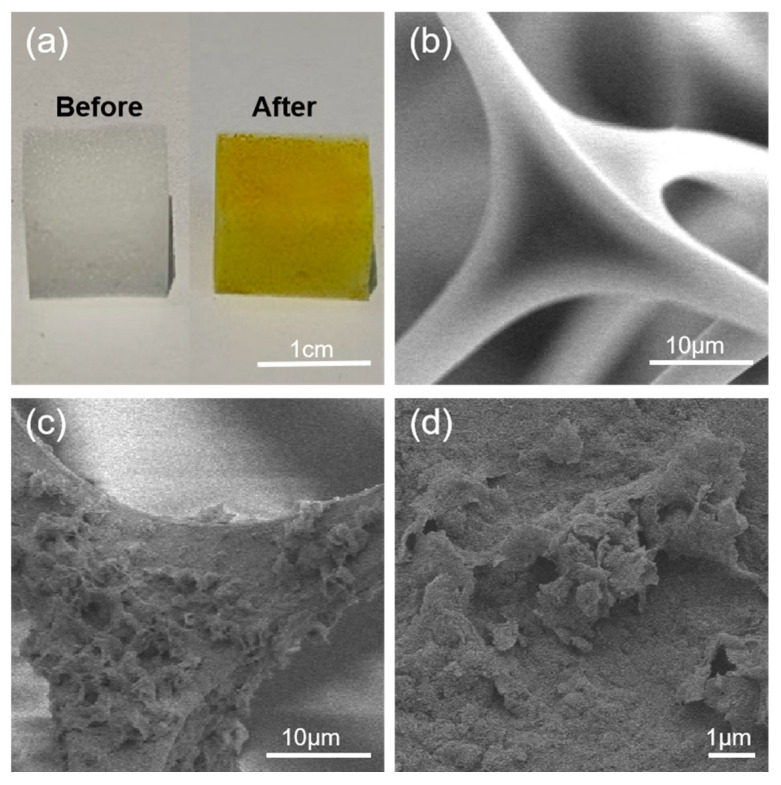
(**a**) Optical images of MS and BD-MS under natural light; (**b**) SEM image of MS; (**c**,**d**) SEM images of BD-MS.

To develop high-performance materials capable of efficiently removing and adsorbing dye molecules from wastewater, we focused on leveraging the abundant amino groups on the surface of MS, which provide multiple anchoring sites for grafting 2,2′-bipyridine-5,5′-dicarboxaldehyde. By catalyzing the Schiff base reaction between benzidine and BPA with acetic acid at room temperature, a linear polymer network was constructed on the MS surface, yielding a fully BD-MS, as illustrated in Scheme S1. Notably, the BD-MS exhibited significantly enhanced adsorption capacity compared to the MS (Figure S1). Although the initial synthetic protocol achieved surface modification via a solvent-mediated reaction at room temperature, the 24 h reaction time was not only prolonged but also resulted in incomplete surface coverage and limited polymer grafting, leading to suboptimal adsorption performance (Figure S2). Systematic optimization reveals that elevating the reaction temperature substantially accelerated reaction kinetics, enabling complete surface modification within a reduced timeframe while increasing polymer grafting density. As clearly demonstrated in Figure 2a, the unmodified melamine sponge exhibited an overall white morphology with abundant macroporous structures. After coating with the linear polymer network, the sponge turned uniformly yellow. SEM images (Figure 2b) confirm that the pristine sponge skeleton consisted of interconnected fibrous structures with exceptionally smooth surfaces. Following modification, BD-MS retained its interconnected fibrous architecture but uniformly developed a unique nano-scale petal-like morphology on the framework (Figure 2c,d), forming a microstructured rough surface. This structural evolution substantially increased accessible active sites, which is critical for rapid mass transfer during CR adsorption.

**Figure 3:**
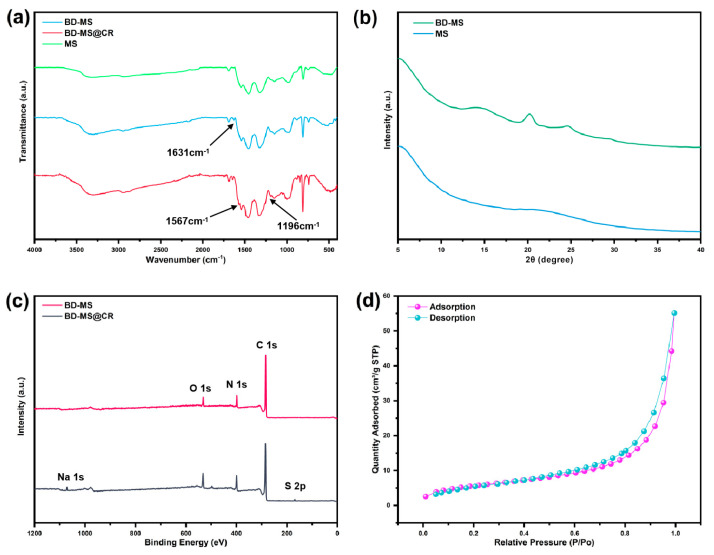
(**a**) FTIR spectra of MS, BD-MS, and BD-MS@CR; (**b**) XRD patterns of MS and BD-MS; (**c**) XPS spectra of BD-MS and BD-MS@CR; (**d**) N_2_ adsorption–desorption isotherm of BD-MS.

The successful grafting of linear polymer networks onto the melamine sponge was systematically verified through spectroscopic and crystallographic analyses. As illustrated in the FTIR spectrum (Figure 3a), the modified sponge exhibited a prominent C=N stretching vibration peak at 1631 cm^−1^, absent in pristine MS. This characteristic peak confirms the formation of imine bonds on the sponge framework via the Schiff base reaction between BD and BPA [35]. Further evidence arises from the redshifted and intensified N-H stretching vibration, shifting from 3322 cm ^−1^ in MS to 3295 cm^−1^ in BD-MS, attributable to residual free amino groups in the grafted polymer network. Crucially, the modified MS matrix retained characteristic absorption peaks of triazine rings at 808 cm^−1^, 1330 cm^−1^, 1455 cm^−1^, and 1545 cm^−1^, corresponding to in-plane deformation modes and C=N stretching/torsional vibrations, thereby confirming structural integrity post-modification [19]. XRD patterns (Figure 3b) provided complementary structural insights. Both MS and BD-MS displayed broad amorphous humps rather than sharp crystalline peaks, consistent with their foam-derived architectures. The absence of higher-crystallinity linear polymer networks on BD-MS stems from π-π interactions between BD and BPA [36]. Enhanced low-angle scattering (<5°) originated from the sponge matrix’s intrinsic nanoscale porosity. Notably, MS preserved this scattering signature, verifying retention of the nanoscale porous framework. However, BD-MS exhibited distinct amorphous broadening features at 20.2° and 24.6°, corresponding to grafted polymer phases. This contrast confirms successful polymer integration without compromising the sponge’s inherent porous architecture [37].

TG analysis reveals the thermal stability of the materials (Figure S3). Pristine MS underwent significant thermal decomposition at 392 °C, with a mass loss of 44%. After linear polymer modification, BD-MS exhibited a comparable thermal decomposition onset (394 °C) but demonstrated a markedly reduced mass loss by 10 percentage points, indicating substantially enhanced thermal stability. Notably, BD-MS showed only 13% mass loss below 354 °C, primarily attributed to the evaporation of residual moisture and organic solvents. A secondary thermal decomposition event observed at 521 °C for BD-MS originated from the cleavage of surface-grafted polymer chains. These data provide compelling evidence for the exceptional thermal stability of BD-MS, underpinning its reliable performance as an adsorbent for real-world deployment. Based on its predominantly macroporous structure and negligible N_2_ adsorption capacity, MS cannot achieve a measurable BET specific surface area. Nitrogen adsorption–desorption isotherms measured at 77 K indicate a significantly improved microporous structure in BD-MS. The material exhibits a BET specific surface area of 20.80 m^2^/g (Figure 3d), while its average pore size, calculated using the BJH model from the data in Figure S4, is 16.4 nm. The hierarchical porous structure integrating micropores and interconnected macropores synergizes with the material’s inherent superhydrophilicity. This architecture not only provides efficient diffusion pathways for dye molecules but also amplifies interfacial interactions between surface-active sites and dyes through enhanced specific surface area. The microporous–macroporous composite system in BD-MS facilitates mass transfer efficiency, while the high surface area ensures abundant active sites for adsorption. Coupled with improved thermal stability that guarantees structural integrity under elevated temperatures, these characteristics collectively highlight BD-MS’s exceptional potential for dye adsorption/separation in wastewater treatment applications.

### 3.2. Adsorption Isotherms of Dye on BD-MS

**Figure 4:**
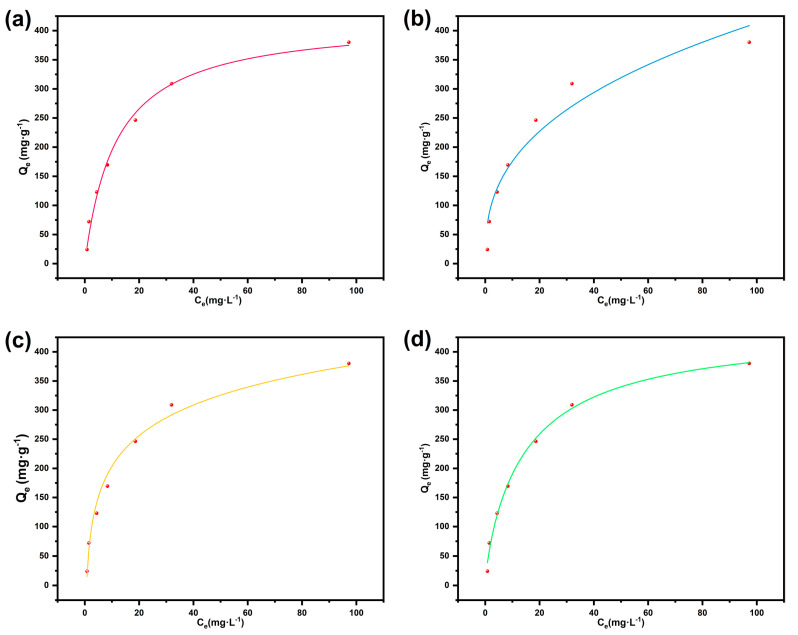
Adsorption isotherm models for BD-MS toward Congo red: (**a**) Langmuir model; (**b**) Freundlich model; (**c**) Temkin model; (**d**) Sips model.

To evaluate the adsorption performance of BD-MS toward CR dye in wastewater, systematic adsorption isotherm experiments were conducted. Under dark conditions at room temperature, quantified BD-MS samples were immersed in CR solutions of varying initial concentrations. After reaching adsorption equilibrium over 72 h, the quantitative relationship between Q_e_ and C_e_ was determined (Figure 4). The experimental results reveal that Q_e_ initially increased rapidly with rising initial concentrations before plateauing, attributed to the rapid occupation of limited adsorption sites at lower concentrations. The measured Q_m_ reached 380.4 mg·g^−1^. Two classical isotherm models, the Langmuir and the Freundlich adsorption models, were applied to fit the data using nonlinear regression. The fitting parameters are summarized in Table S1. Both models exhibited correlation coefficients above 0.9, indicating good fitting quality. The Langmuir model provided a marginally superior fit compared to the Freundlich model, suggesting that monolayer adsorption on homogeneous BD-MS surfaces dominated the process, with negligible interactions between adsorbed molecules. The Langmuir-derived Q_m_ (403.8 mg g^−1^) closely matched the experimental value. However, the potential contribution of heterogeneous surfaces and multilayer adsorption cannot be entirely ruled out, as supported by the strong fitting to the Sips equation—a hybrid of the Langmuir and Freundlich models. Furthermore, effective fitting to the Temkin model implied a temperature-dependent, exothermic adsorption process [38]. These findings demonstrate that the BD-MS composite, combining the inherent porous architecture of MS with uniformly grafted linear polymer networks, provides abundant homogeneous adsorption sites, although its surface is not perfectly homogeneous, and interactions between adsorbate molecules or the potential for multilayer coverage may exist. This structural synergy endows BD-MS with significant application potential in dye-laden wastewater treatment.

### 3.3. Adsorption Kinetics Profiles

**Figure 5:**
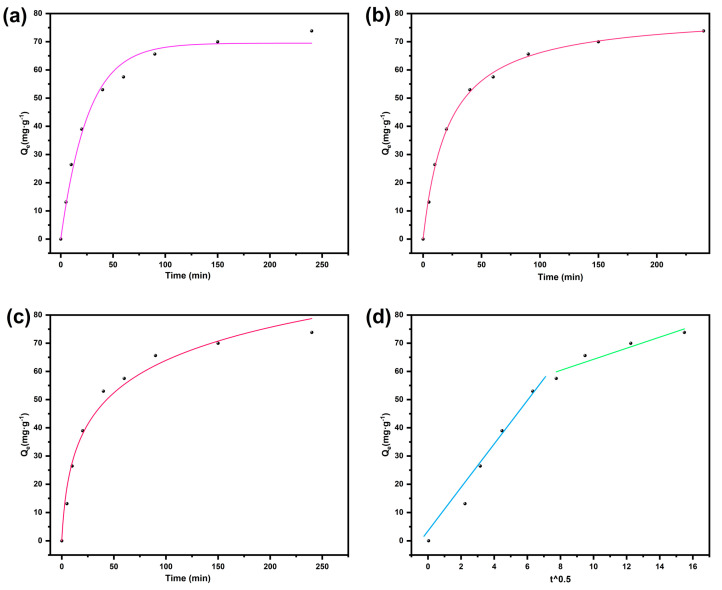
Kinetic models for the adsorption of Congo red onto BD-MS: (**a**) pseudo-first-order; (**b**) pseudo-second-order; (**c)** Elovich; (**d**) intra-particle diffusion model.

To elucidate the adsorption kinetic mechanism of BD-MS for CR dye, this study systematically investigated the influence of contact time on adsorption capacity under low-concentration conditions (40 mg·L^−1^) (Figure 5). Through nonlinear fitting of experimental data using pseudo-first-order, pseudo-second-order, Elovich, and intraparticle diffusion models (Table S2), it was found that the pseudo-second-order kinetic model optimally described the adsorption process. The validity of this model indicates that the adsorption process involves chemisorption, from which it can be inferred that the adsorption behavior of CR on BD-MS samples may involve chemical adsorption through valence forces via electron sharing or exchange [33]. The intraparticle diffusion model revealed two distinct stages prior to reaching adsorption equilibrium, corresponding, respectively, to the transport of dye molecules from the solution to the external surface of the adsorbent and from the external surface to the interior of BD-MS. The first stage exhibited a higher slope, indicating rapid occupation of BD-MS surface and macropores in the sponge matrix during the initial adsorption phase. This rapid stage can be attributed to the linear structure of CR (Figure S5), which facilitates its easy entry into BD-MS pores, thereby enhancing CR adsorption. The second stage was characterized by a significantly reduced slope, resulting from hindered diffusion of adsorbate into adsorbent pores. The fitted straight line for BD-MS did not pass through the origin, suggesting the coexistence of multiple mechanisms in the CR adsorption process, potentially governed by simultaneous intraparticle diffusion, pore diffusion, and surface adsorption [39]. In conclusion, the adsorption kinetics and intraparticle diffusion model demonstrate that CR adsorption on BD-MS is predominantly chemisorption-driven, with the internal pore structure of BD-MS exerting a notable influence on the adsorption process.

### 3.4. Adsorption Mechanism

**Figure 6:**
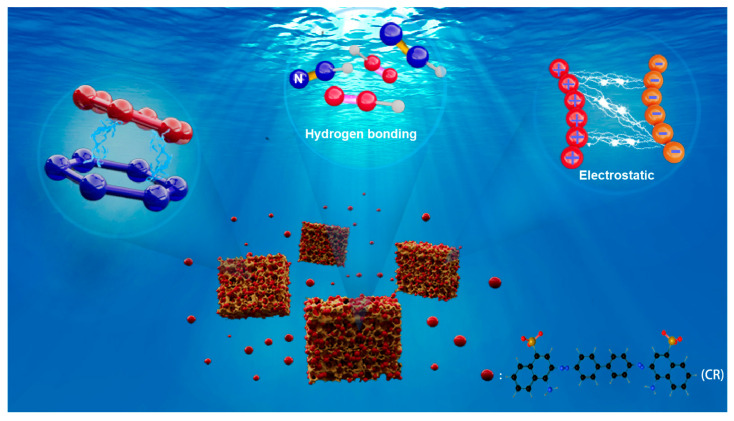
Adsorption mechanism of Congo red by BD-MS.

To elucidate the adsorption mechanism, XPS spectra of BD-MS after CR adsorption were recorded (Figure 3c). Distinct Na 1s and S 2p peaks were observed, confirming the successful adsorption of CR molecules. Comparative FTIR analysis of BD-MS before and after CR adsorption (Figure 3a) reveals new peaks at 1567 cm^−1^ and 1196 cm^−1^, attributed to the bending vibration of N=N and symmetric stretching of –SO_3_^−^ groups in CR, respectively. These spectral features demonstrate potential interactions between BD-MS and CR, corroborating effective adsorption. XPS and FT-IR analyses further indicate the presence of abundant nitrogen-containing functional groups (C=N, C–N, and –NH_2_) and oxygen-containing groups (–C=O) in BD-MS. CR, rich in amino (–NH_2_) and sulfonate (–SO_3_^−^) groups, forms N–H···N and O–H···N hydrogen bonds with BD-MS, serving as primary adsorption driving forces. The C=N stretching vibration shifted from 1631 cm^−1^ to 1640 cm^−1^, attributed to hydrogen bonding interactions between BD-MS and CR. In acidic solutions, protonation of amino groups (–NH_2_ → –NH_3_^+^) on BD-MS and dissociation of sulfonate groups (–SO_3_H → –SO_3_^−^ + H^+^) on CR suggest electrostatic interactions that enhance adsorption. Additionally, the conjugated aromatic structures in BD-MS and CR enable π-π stacking between their aromatic rings, further strengthening CR adsorption [40]. The synergistic effects of hydrogen bonding, electrostatic interactions, and π-π stacking collectively contribute to the exceptional adsorption capacity of BD-MS for CR (Figure 6). This multi-mechanistic framework underscores the material’s potential for efficient dye removal in wastewater treatment applications.

### 3.5. Adsorption Performance and pH-Dependent Adsorption Efficiency

**Figure 7:**
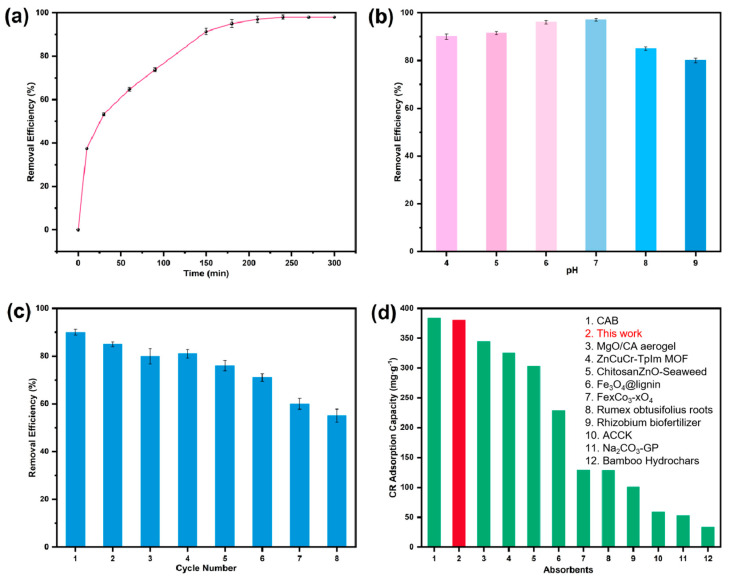
Adsorption performance of BD-MS for Congo red: (**a**) time-dependent removal efficiency; (**b**) pH-dependent removal efficiency; (**c**) removal efficiency under regeneration cycles; (**d**) comparative maximum adsorption capacity with other adsorbents.

As shown in Figure 7a, BD-MS exhibits exceptional adsorption kinetic characteristics for CR. In a CR solution with an initial concentration of 40 m L^−1^, the adsorbent achieved adsorption equilibrium within 4 h, demonstrating a high removal efficiency of 98%. Its outstanding removal efficiency and rapid equilibrium attainment highlight BD-MS’s superior potential for wastewater treatment. Solution pH plays a critical role in the adsorption process, as pH-dependent surface charge variations influence the dissociation of functional groups at active sites, thereby modulating adsorption capacity. Figure 7b demonstrates that while adsorption capacity decreases to varying degrees with pH deviations from neutrality, the removal rate remains consistently high, confirming the adsorbent’s excellent chemical stability across acidic/alkaline environments. Under alkaline conditions, increased OH^−^ ion concentration imparts a negative surface charge on BD-MS, generating electrostatic repulsion with the anionic CR dye. More importantly, CR competes intensively with OH^−^ ions for active sites, leading to reduced adsorption efficiency compared to acidic conditions. In acidic media, although H^+^ ions may partially disrupt functional groups containing adsorption sites, the strong electrostatic attraction between protonated amino groups on BD-MS and dissociated-SO_3_^−^ groups of CR maintains high adsorption capability.

### 3.6. Repeatability and Adsorption of Other Dyes

Reusable materials not only enhance economic benefits but also reduce environmental impacts, making the recyclability of adsorbents crucial for practical applications. To achieve BD-MS regeneration, 95% ethanol was utilized to immerse CR-saturated BD-MS for 24 h, effectively desorbing CR and reactivating the adsorbent. As shown in Figure 7c, the Congo red removal rate by the BD-MS remained above 50% after eight consecutive cycles. The decline in adsorption performance may stem from two aspects: First, the repeated regeneration processes could compromise the porous structure and active sites of the material. Second, some dye molecules may form strong chemical bonds with the adsorbent, preventing their full desorption during the regeneration processes, thereby compromising subsequent adsorption performance. These results demonstrate BD-MS’s effectiveness in CR adsorption from aqueous solutions, with advantages including cost-effectiveness, facile regeneration, and high stability, positioning it as a promising and sustainable candidate for real-world wastewater treatment and dye removal. To comprehensively evaluate the adsorption applicability of BD-MS toward diverse organic dyes, representative cationic dyes (malachite green, MG; rhodamine B, RhB; and methylene blue, MB) and an anionic dye (methyl orange, MO) were selected. Experimental results (Figure S6) demonstrate that BD-MS exhibits exceptional adsorption performance across all tested dyes. For the cationic dyes, MG showed the highest removal efficiency at 80%, followed by RhB at 54%, while MB displayed the lowest efficiency at 32%. Remarkably, the anionic dye MO achieved a removal efficiency as high as 57%. This selective adsorption originates primarily from multiple molecular interactions between BD-MS and the dye molecules. The π-π interactions between the aromatic ring structures of BD-MS and the dyes were crucial for the adsorption process, enabling the tight binding of dye molecules to the sponge surface. Additionally, the oxygen- and nitrogen-containing functional groups on BD-MS formed hydrogen bonds with the dye molecules, further enhancing the adsorption efficiency. Notably, electrostatic interactions also played a role in the adsorption process [41]. The synergistic effect of these multi-mechanistic interactions underpinned BD-MS’s ability to efficiently adsorb various dyes. Quantitative comparative analysis of maximum adsorption capacities with other composite materials (full data in Table S3) reveals that BD-MS outperforms most reported dye adsorbents (Figure 7d).

## 4. Conclusions

In summary, this study successfully constructed BD-MS with a surface-grafted linear polymer network via Schiff base condensation between benzidine and 2,2′-bipyridine-5,5′-dicarbaldehyde under solvothermal conditions. The material demonstrated exceptional adsorption performance for CR, achieving a maximum adsorption capacity of 380.3 mg·g^−1^ through synergistic π-π stacking, hydrogen bonding, and electrostatic interactions, with adsorption equilibrium attained within 4 h. BD-MS also exhibited high adsorption efficiency toward multiple dyes. The adsorption process for CR on BD-MS was best described by the Langmuir isotherm and pseudo-second-order kinetic models. Stability evaluations confirmed BD-MS’s superior thermal, chemical, and mechanical robustness. After eight adsorption–desorption cycles, CR removal efficiency remained above 50%, highlighting its excellent regenerability. This work proposes a novel paradigm for developing efficient and stable dye wastewater treatment materials through hierarchical structural design and multi-mechanistic synergistic strategies.

## Data Availability

Data is contained within the article or Supplementary Materials.

## Supplementary Materials

The following supporting information can be downloaded at: https://www.mdpi.com/article/10.3390/nano15151157/s1, Table S1: Parameters of isotherm modes on BD-MS; Table S2: Parameters of kinetics isotherm modes on BD-MS; Table S3: Comparison of CR adsorption capacity in various composites. Figure S1: Adsorption of Congo red solution by (a) MS and (b) BD-MS; Figure S2: SEM images of BD-MS synthesized at room temperature; Figure S3: (a) TGA and (b) DTG curves of MS and BD-MS; Figure S4: N_2_ adsorption isotherms and pore size distribution of BD-MS (77K); Figure S5: Molecular structures of different dye compounds; Figure S6:UV-Vis spectra of different dyes adsorbed by BD-MS. (a) Rhodamine B, (b) malachite green, (c) methylene blue, (d) methyl orange; Scheme S1: Preparation of BD-MS. Refs. [8,9,10,42,43,44,45,46,47,48,49] are cited in the Supplementary Materials File.
