# Supplementary material for: Schiff Base-Functionalized Melamine Sponge with Hierarchical Porous Architecture for High-Efficiency Removal of Organic Dyes in Wastewater
Source: Nanomaterials (Basel). 2025 Jul 26;15(15):1157. doi: 10.3390/nano15151157 (PMC12348324; doi:10.3390/nano15151157)
Supplement: Supplementary file 1 [file nanomaterials-15-01157-s001.zip › nanomaterials-3751748-supplementary.pdf]

Supporting Information

Schiff Base-Functionalized Melamine Sponge with Hierarchical Porous Architecture for High-Efficiency Removal of Organic Dyes in Wastewater

Xiaoyu Du [1], Hailiang Nie [1], Yanqing Qu [1*], Jingyu Xu [1], Hongge Jia [1], Yong Zhang [1], Wenhui Ma [1], Boyu Du [2*]

1 College of Chemistry and Chemical Engineering, Heilongjiang Provincial Key Laboratory of Polymeric composition, Qiqihar University, Qiqihar, Heilongjiang 161006, China

2 Guangxi Key Laboratory of Clean Pulp & Papermaking and Pollution Control, Guangxi University, Nanning 530004, China

* Correspondence: vipquyanqing@163.com; duboyu@gxu.edu.cn

Table of Contenes

SECTION A. SUPPORTING SCHEME S2
SECTION B. SUPPORTING FIGURES S2
SECTION C. SUPPORTING TABLES S5
SECTION D. SUPPORTING REFERENCES S7

Section A. Supporting Scheme

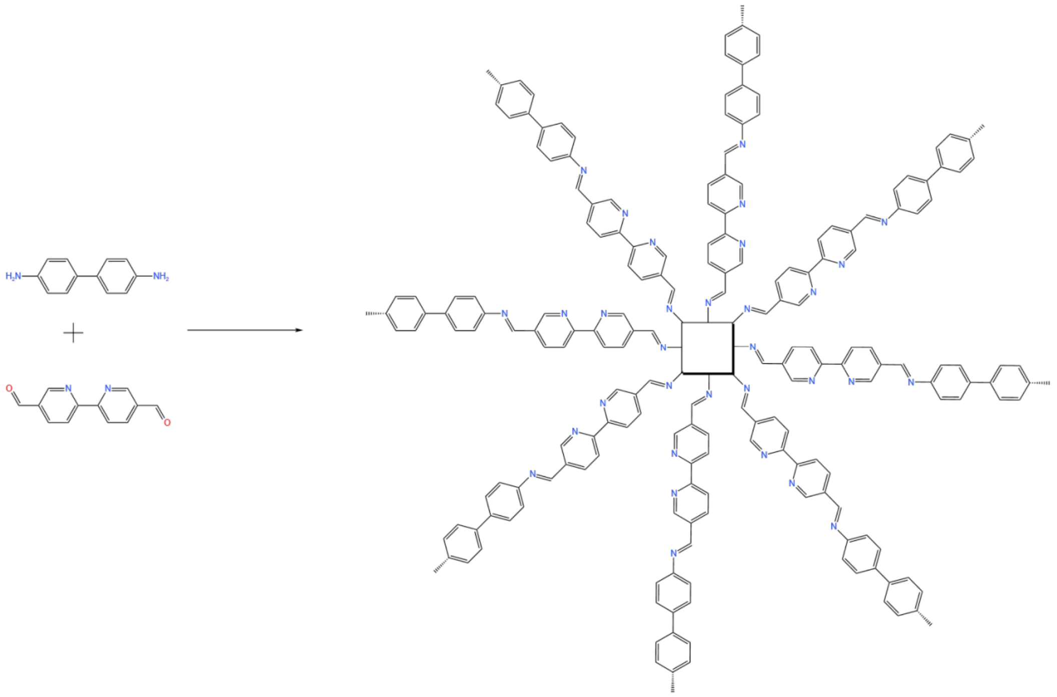

Scheme S1. Preparation of BD-MS.

Section B. Supporting Figures

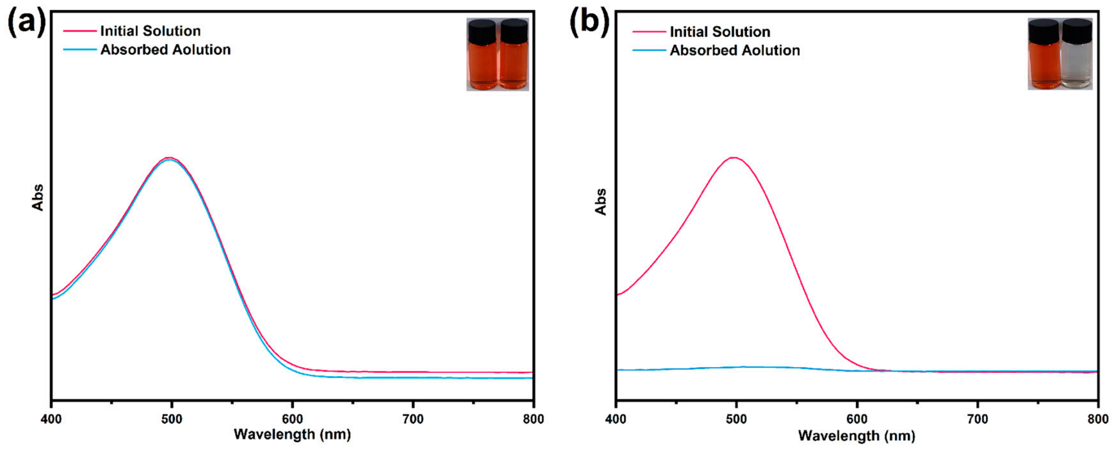

Figure S1. Adsorption of Congo red solution by (a) MS and (b) BD-MS

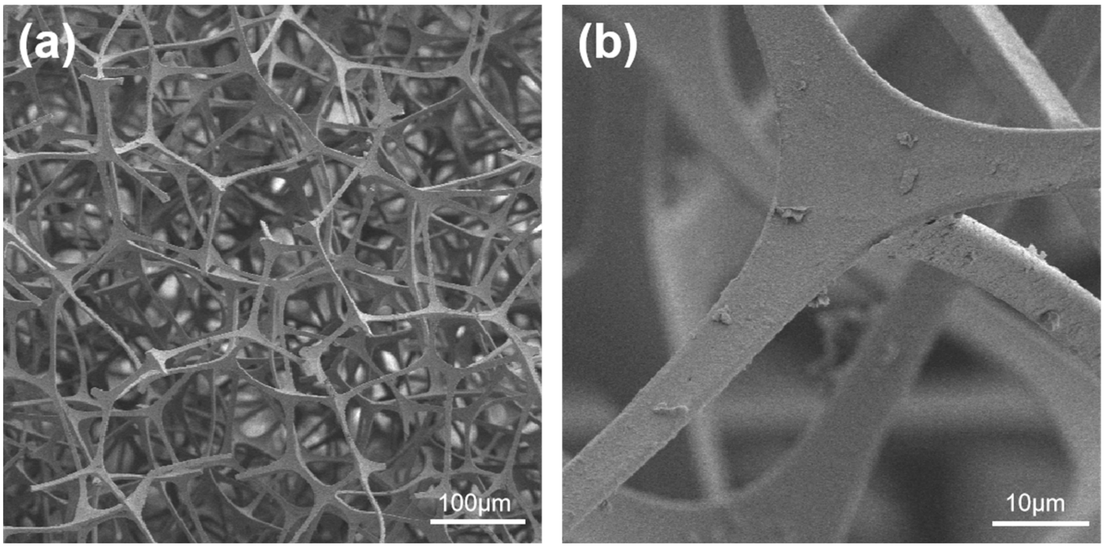

Figure S2. SEM images of BD-MS synthesized at room temperature.

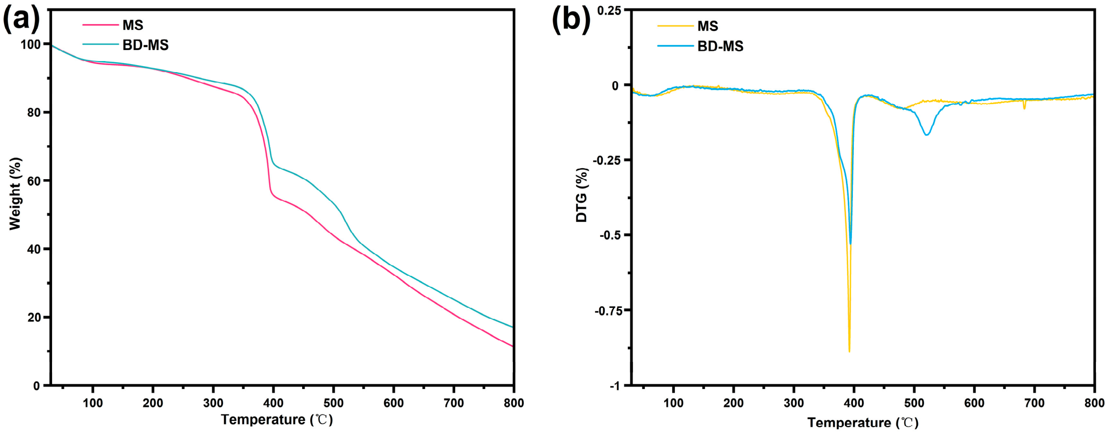

Figure S3. (a) TGA and (b) DTG curves of MS and BD-MS

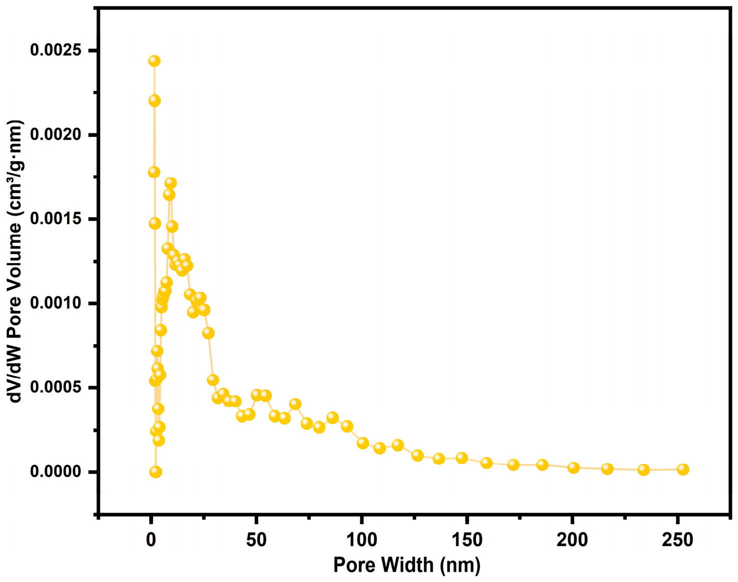

Figure S4. Pore size distribution of BD-MS (77K).

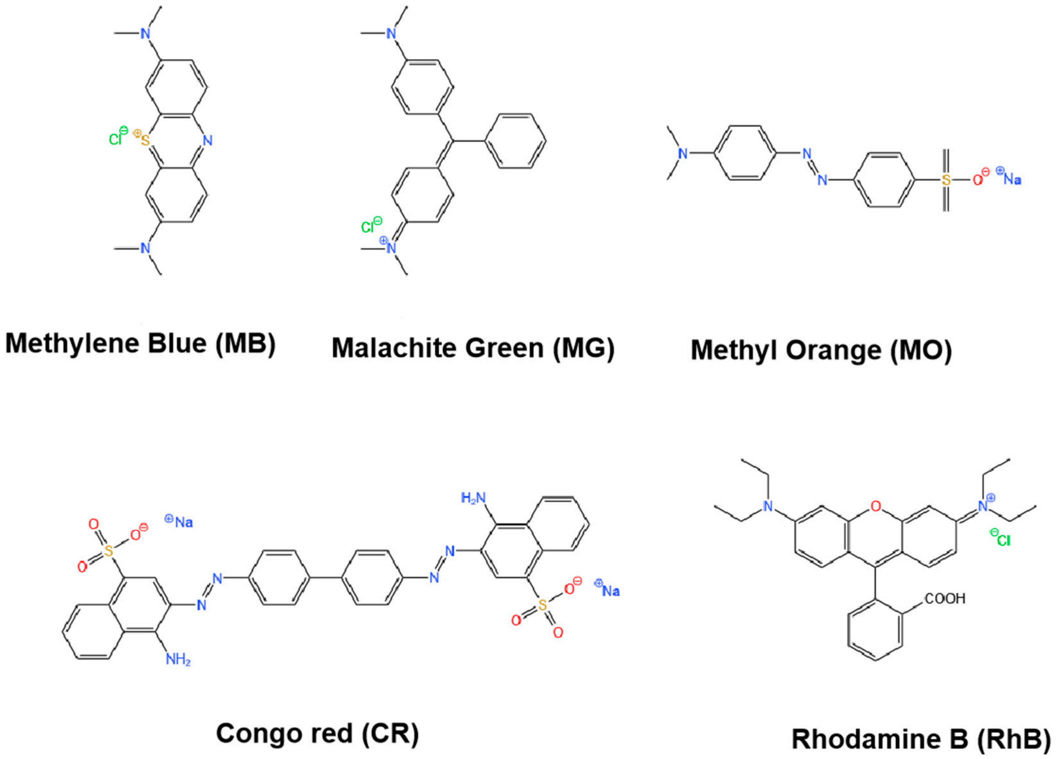

Figure S5. Molecular structures of different dye compounds.

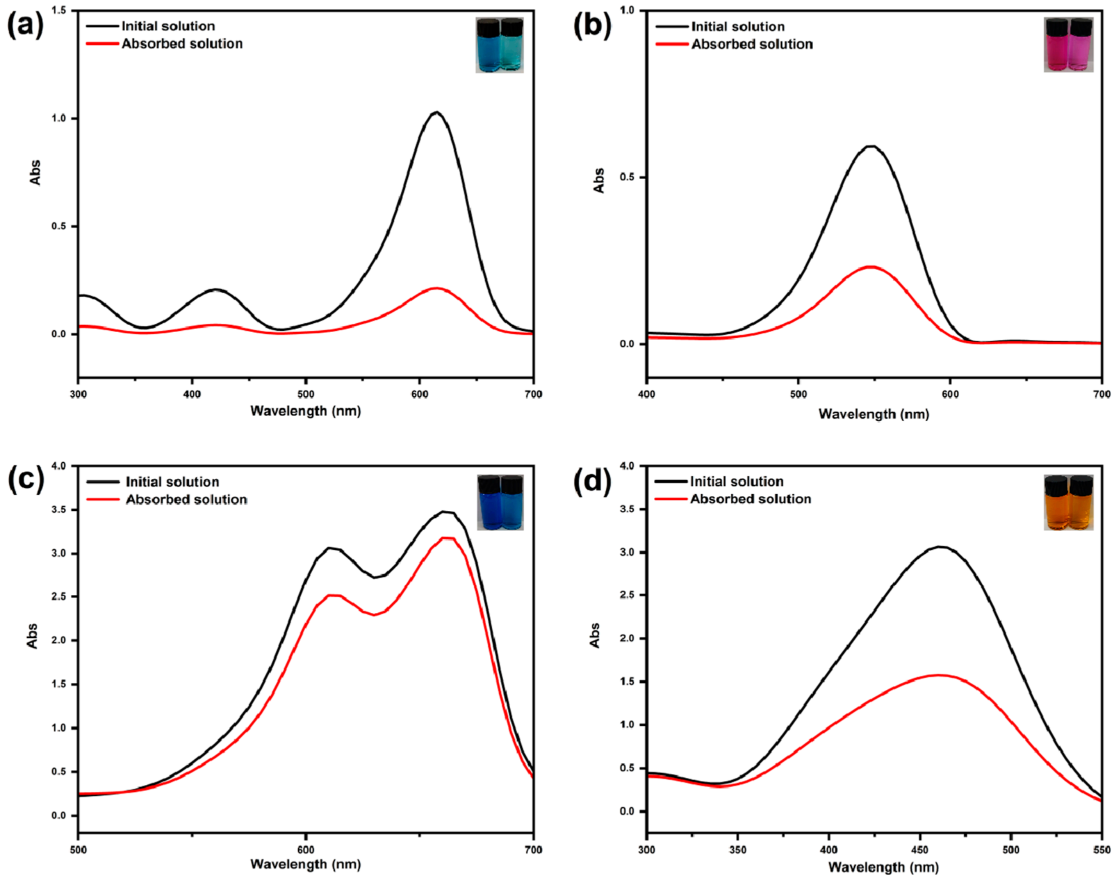

Figure S6. UV-Vis spectra of different dyes adsorbed by BD-MS. (a) Malachite Green, (b) Rhodamine B, (c) Methylene Blue, (d) Methyl Orange.

Section C. Supporting Tables

Table S1. Parameters of Isotherms isotherm modes on BD-MS.

Models	Parameters	CR
Langmuir	Q_m ($mg \cdot g^{-1}$)	403.8
	K_L	0.08
	R^2	0.98827
Freundlich	K_F	74.58
	R^2	0.94681
Temkin	B	75.87
	A	1.46
	R^2	0.96693
Sips	Q_m ($mg \cdot g^{-1}$)	441.17
	K_S	0.74

	R^2	0.98518

Table S2. Parameters of Kinetics isotherm modes on BD-MS.

Models	Parameters	CR
Pseudo-first order	K_1	0.038
	R^2	0.94326
Pseudo-second order	K_2	80.403
	R^2	0.99815
Elovich	α	6.871
	β	0.058
	R^2	0.97023
Intraparticle	K_1	7.944
	K_2	1.967
	R_1^1	0.951
	R_2^2	0.875

Table S3. Comparison of CR adsorption capacity in various composites.

Dye	Materials	Q_m (mg g^{-1})	Ref.
CR	CAB	384	[1]
	BD-MS	380.4	This work
	MgO/CA aerogel	344.8 (323K)	[2]
	ZnCuCr-TpIm MOF	325	[3]
	ChitosanZnO-Seaweed	303.03	[4]
	Fe_3O_4@lignin	229	[5]
	FexCo3-xO_4	128.6	[6]
	Rumex obtusifolius roots	128.21	[7]
	Rhizobium biofertilizer	101.01	[8]
	ACCK	59.11	[9]
	Na_2CO_3-GP	52.6	[10]
	bamboo hydrochars	33.7	[11]

Section D. Supporting References

1. Zhang, H.; Zhou, J.; Muhammad, Y.; Tang, R.; Liu, K.; Zhu, Y.; Tong, Z. Citric Acid Modified Bentonite for Congo Red Adsorption. *Front. Mater.* **2019**, *6*, 5, doi:10.3389/fmats.2019.00005.
2. Cui, M.; Li, Y.; Sun, Y.; Wang, H.; Li, M.; Li, L.; Xu, W. Study on Adsorption Performance of MgO/Calcium Alginate Composite for Congo Red in Wastewater. *J. Polym. Environ.* **2021**, *29*, 3977–3987, doi:10.1007/s10924-021-02170-x.
3. Al-Omari, M.H.; Abu-Rayyan, A.; H. Ragab, A.; A. Taher, M.; M. El-Sayed, E.-S.; Elfiky, A.; Taha, A.; Mubarak, M.F. Optimized Congo Red Dye Adsorption Using ZnCuCr-Based MOF for Sustainable Wastewater Treatment. *Langmuir* **2025**, *41*, 5947–5961, doi:10.1021/acs.langmuir.4c04661.
4. Gopalakrishnan, S.; Kannan, P.; Balasubramani, K.; Rajamohan, N.; Rajasimman, M. Sustainable Remediation of Toxic Congo Red Dye Pollution Using Bio Based Carbon Nanocomposite: Modelling and Performance Evaluation. *Chemosphere* **2023**, *343*, 140206, doi:10.1016/j.chemosphere.2023.140206.
5. Fang, L.; Wu, H.; Shi, Y.; Tao, Y.; Yong, Q. Preparation of Lignin-Based Magnetic Adsorbent from Kraft Lignin for Adsorbing the Congo Red. *Front. Bioeng. Biotechnol.* **2021**, *9*, doi:10.3389/fbioe.2021.691528.
6. Liu, J.; Wang, N.; Zhang, H.; Baeyens, J. Adsorption of Congo Red Dye on FexCo3-xO4 Nanoparticles. *J. Environ. Manage.* **2019**, *238*, 473–483, doi:10.1016/j.jenvman.2019.03.009.
7. Isik, B. Adsorptive Removal of Congo Red and Methylene Blue Dyes from Aqueous Solutions by Rumex Obtusifolius Roots. *Korean J. Chem. Eng.* **2023**, *40*, 3003–3016, doi:10.1007/s11814-023-1569-x.
8. Kulkarni, K.; Kurhade, S.; Chendake, Y.; Kulkarni, A.; Satpute, S. Utilization of Low Cost Biofertilizers for Adsorptive Removal of Congo Red Dye. *Bull. Environ. Contam. Toxicol.* **2023**, *111*, 33, doi:10.1007/s00128-023-03784-8.
9. He, J.; Zhu, L.; Guo, S.; Yang, B. An Effective Strategy for Coal-Series Kaolin Utilization: Preparation of Magnetic Adsorbent for Congo Red Adsorption. *Chem. Eng. Sci.* **2025**, *304*, 120958, doi:10.1016/j.ces.2024.120958.
10. Pulikkal, A.K.; Laskar, N.; Anjudikkal, J. Effective Adsorption of Polycyclic Aromatic Congo Red Dye by Modified Garlic Peel. *J. Dispers. Sci. Technol.* **2023**, *25*, 799–809, doi:10.1080/01932691.2023.2181180.
11. Li, Y.; Meas, A.; Shan, S.; Yang, R.; Gai, X. Production and Optimization of Bamboo Hydrochars for Adsorption of Congo Red and 2-Naphthol. *Bioresour. Technol.* **2016**, *207*, 379–386, doi:10.1016/j.biortech.2016.02.012.
